# Cause of Death Affects Racial Classification on Death Certificates

**DOI:** 10.1371/journal.pone.0015812

**Published:** 2011-01-26

**Authors:** Andrew Noymer, Andrew M. Penner, Aliya Saperstein

**Affiliations:** 1 Department of Sociology, University of California Irvine, Irvine, California, United States of America; 2 Department of Population Health and Disease Prevention, University of California Irvine, Irvine, California, United States of America; 3 Health and Global Change Project, International Institute for Applied Systems Analysis, Laxenburg, Austria; 4 Department of Sociology, University of Oregon, Eugene, Oregon, United States of America; National Institutes of Health, United States of America

## Abstract

Recent research suggests racial classification is responsive to social stereotypes, but how this affects racial classification in national vital statistics is unknown. This study examines whether cause of death influences racial classification on death certificates. We analyze the racial classifications from a nationally representative sample of death certificates and subsequent interviews with the decedents' next of kin and find notable discrepancies between the two racial classifications by cause of death. Cirrhosis decedents are more likely to be recorded as American Indian on their death certificates, and homicide victims are more likely to be recorded as Black; these results remain net of controls for followback survey racial classification, indicating that the relationship we reveal is not simply a restatement of the fact that these causes of death are more prevalent among certain groups. Our findings suggest that seemingly non-racial characteristics, such as cause of death, affect how people are racially perceived by others and thus shape U.S. official statistics.

## Introduction

The accuracy of official data on birth rates and death rates are often taken for granted. However, recent research has drawn attention to inconsistencies in the recording of race across data sources and the resulting variability in estimates of race-specific death rates in the United States [1], [2]. These analyses have sparked debate among researchers over which measure of race should be considered correct [3]. Rather than focus on identifying errors or inaccuracies in the data, we extend previous research by exploring how the discrepancies in race reporting arise and whether they provide insight into why racial disparities in vital statistics persist. In particular, we use a nationally representative sample of death certificates and matched data from a subsequent survey of the decedent's next of kin to examine whether cause of death and other non-racial characteristics of decedents are related to their racial classification.

According to official statistics for the period under study, American Indians were 2.6 times more likely to die of cirrhosis or chronic liver disease than Whites, and Blacks were 6.6 times more likely than Whites to be victims of homicide in the United States [4]. Drawing from previous research on how medical examiners make decisions under conditions of uncertainty [5], as well as work demonstrating a relationship between social status and racial classification [6], we postulate that these well-known racial disparities in cause of death may unintentionally become self-fulfilling prophecies as medical examiners, funeral directors and others make decisions about how to complete an individual's death certificate. In addition to calling into question the interpretation of race and cause-specific death rates in the United States, our results suggest that racial stereotypes are one mechanism shaping how people are perceived and classified by others.

## Results

Echoing previous findings of inconsistencies in how an individual is racially classified across different data sources, we find that for 1.1 percent of death certificates in the 1993 National Mortality Followback Survey, the next of kin racially classify the decedent differently than was recorded in official statistics. Levels of inconsistency range from 1 percent among decedents classified as white on their death certificate to 8.8 percent among decedents classified as American Indians.

We build on this finding by estimating the effect of cause of death on death certificate racial classification in two sets of logistic regression models (Table 1). Our focus in this study is not on the level of inconsistency in racial classification, per se, but on assessing whether the discrepancies are patterned according to other characteristics of the decedent. In the first set of models, we examine whether the decedent was classified as American Indian on the death certificate as a function of whether the decedent died of cirrhosis (Table 1A); the second set of models (5–8) is structured in the same way as the first, but examines the relationship between being the victim of a homicide and being classified as Black on one's death certificate (Table 1B).

**Table 1:** The effect of cause of death on death certificate racial classification.

Panel A. Odds ratios for classification as American Indian as a function of cirrhosis				
	Model 1	Model 2	Model 3	Model 4
Chronic liver disease and cirrhosis	2.89**	2.52*	2.45*	3.53**
	(3.17)	(2.31)	(2.08)	(2.82)
Race and Ethnicity (FS)	X	X	X	X
Demographic characteristics (DC)		X	X	X
Other characteristics (DC)			X	X
Other characteristics (FS)				X
Model degrees of freedom	6	57	80	162
N	22794	22524	22508	19531

Panel B. Odds ratios for classification as Black as a function of homicide				
	Model 5	Model 6	Model 7	Model 8
Homicide and legal intervention	2.45***	2.97***	5.83***	4.43***
	(11.80)	(12.30)	(15.00)	(11.20)
Race and Ethnicity (FS)	X	X	X	X
Demographic characteristics (DC)		X	X	X
Other characteristics (DC)			X	X
Other characteristics (FS)				X
Model degrees of freedom	6	59	83	184
N	22794	22747	22747	22747

Note: *** p<0.001, ** p<0.01, * p<0.05, two tailed test. z statistics in parentheses.

FS indicates followback survey, DC indicates death certificate. All models contain controls for FS race and ethnicity. When the number of cases used to estimate each model changes between models it is because some independent variables predict the death certificate racial classification perfectly (e.g. there were no American Indians in certain occupational or income categories). Estimating all models using the smallest sample size does not substantively change the results (results available in online supplementary Materials S1).

The baseline model examining the relationship between dying of cirrhosis and one's racial classification indicates that the odds of being classified as American Indian were 2.9 times higher for people who died of cirrhosis (Table 1A). This model holds constant the race reported by the next of kin on the followback survey, which accounts for the fact that cirrhosis is a more prevalent cause of death among American Indians. As the relationship between dying of cirrhosis and being classified as American Indian on one's death certificate remains statistically significant after we introduce additional controls (Models 2–4), we conclude that the effect of cirrhosis mortality on racial classification is not attributable to any other measured factors, such as the decedent's income or region of residence. Moreover, we see that introducing these controls increases the effect of cirrhosis on the odds of being classified as American Indian, demonstrating that this effect only gets stronger when we are comparing otherwise similar decedents.

The second set of models reveals a similar pattern for the effect of being a homicide victim on the odds of being classified as Black on the death certificate (Table 1B). The similarity of these findings to the results for American Indians is surprising – both because of the lower rates of racial reporting inconsistency for Blacks (1.1 percent) and the historically more rigid rules for being classified as Black in the United States [7]. When we control only for followback survey race, we find that the odds of being classified as Black on the death certificate were 2.4 times higher for homicide victims (Model 5). Once we introduce controls for additional information from the death certificate and followback survey, we find that the odds that a homicide victim was classified as Black were 4.4 times greater than those who died of other causes (Model 8). Another way to think about the implications of our results is that 204 of the 12,937 Black homicide victims in 1993 official statistics would not have been recorded as Black if they had been classified by their next of kin. Further, we find similar results – in both sets of models – when we examine only non-multiracial, non-Hispanic respondents (as defined by their next of kin), indicating that our findings are not restricted to populations typically thought of as racially ambiguous.

## Discussion

While previous research has demonstrated inconsistencies in racial vital statistics, the processes creating these discrepancies are not well understood. We explored whether seemingly non-racial characteristics of individuals, such as their cause of death, affect how they are perceived racially by others. Our results demonstrate that otherwise similar Americans whose underlying cause of death was chronic liver disease or cirrhosis were more likely to be classified as American Indian on their death certificate than Americans who died of other causes – even if they were not classified as American Indian by their next of kin in a subsequent survey. A similar pattern exists between dying of homicide and the likelihood of being classified as Black. These findings suggest that the racial information recorded in vital statistics may be affected by the same kinds of social processes that shape racial classification more broadly. Research shows that changes in how people are racially classified over their lifetime are related to changes in social status that conform to widely held racial stereotypes [6]. Just as Americans are less likely to be seen as white by a survey interviewer after they have been incarcerated, unemployed or fallen into poverty, we conclude that stereotypes about who is likely to die a particular kind of death may color our official vital statistics.

## Materials and Methods

To explore the role of cause of death in the racial classification of decedents, we examine the most recent public-release data from the National Mortality Followback Survey (NMFS), conducted in 1993. The NMFS matches data from 22,905 death certificates of U.S. residents drawn from the 1993 Current Mortality Sample to information about the decedent gathered from a followback survey using a proxy respondent. The sample is representative of deaths among the 1993 U.S. resident population, age 15 and older, excluding South Dakota – though, importantly for our purposes, homicides were oversampled to provide more statistical power [8]. We drop the 111 cases that are missing information on death certificate race. In 89 percent of the remaining cases, the proxy respondent is the decedent's next-of-kin; we introduce controls for the nature of the relationship into the models, but for the sake of simplicity we refer to the proxy respondent as next-of-kin in discussing the results.

Our key variables are measures of the decedent's race from the death certificate and the followback survey. For both the followback survey and the death certificate, we coded the racial responses into four separate indicators for Black; White; Asian or Pacific Islander; and American Indian, Eskimo, or Aleut. Based on the followback survey, we also created an indicator for whether the decedent was described as “of Spanish or Hispanic origin or descent.” While we would have liked to compare the death certificate race to an earlier racial self-identification by the decedent as well, the NMFS does not include this information, and other publicly available data sources such as the National Longitudinal Mortality Study and the National Health Interview Study Linked Mortality Files keep only one measure of race or use race as a criterion to match respondents to their death certificates. However, the NMFS proxy report of the decedent's race in the followback survey is similar to how many Americans are described by race in the census, where household heads fill in the racial information for all members of the household. Thus, while we lack racial self-identification data, the data we use are comparable to racial data from other official sources.

It is also important to note that we focus on death certificate racial classification in this study because it is used in official statistics. In doing so, we do not mean to imply that the next-of-kin report is the more “true” or correct racial categorization of the decedent. We see all measures of an individual's race as capturing useful information and suggest that examining differences between the measures provides insight into how an individual's race can be defined differently in different contexts. The distribution of next-of-kin racial classifications for the discordant cases is available as online supplementary Materials S1.

In the logistic regressions, our dependent variables reflect the decedent's race as recorded on the death certificate (coded 1 for American Indian, Eskimo, or Aleut, and 0 for all other races in the cirrhosis models, and coded 1 for Black and 0 for all other races in the homicide models). Our primary independent variables are indicators for two causes of death that are commonly reported as being more prevalent among certain racial populations: chronic liver disease and cirrhosis (ICD9 571) among American Indians, and homicide and legal intervention (ICD9 E960–E978, details available as online supplementary Materials S1) among Blacks. These causes were chosen due to well-known racial disparities in the death rates.

We introduce additional controls in sets to test the robustness of the relationship between cause of death and racial classification. In the baseline models, along with cause of death, we include a series of indicator variables for the decedent's race and ethnicity according to the followback survey. The second model in each panel adds demographic characteristics of the decedent reported on the death certificate: age, age-squared, sex, marital status, education, region of birth, month of death, region of death, whether the decedent lived in another state or country and the population size of the decedent's resident county. The third model adds additional death certificate information: the decedent's occupation, veteran status, place of death (e.g. nursing home, hospital), whether an autopsy was conducted and whether the body was referred to a medical examiner. Finally, the fourth model includes all previous controls as well as additional information from the followback survey: the decedent's individual and household income, the proxy respondent's relationship to the decedent, and the proxy respondent's age, gender, and education. Given that our results are consistent across all models and for two different racial populations, we conclude that cause of death is an important predictor of death certificate racial classification.

## Supporting Information

Materials S1 Detailed ICD9 codes, distribution of next-of-kin racial classifications for discordant cases, and models with alternate sample restrictions.
(DOC)
