# Supplementary material for: Cause of Death Affects Racial Classification on Death Certificates
Source: PLoS One. 2011 Jan 26;6(1):e15812. doi: 10.1371/journal.pone.0015812 (PMC3027630; doi:10.1371/journal.pone.0015812)
Supplement: Materials S1 — Detailed ICD9 codes, distribution of next-of-kin racial classifications for discordant cases, and models with alternate sample restrictions. (DOC) [file pone.0015812.s001.doc]

Supplemental information for PLoS ONE article: 10.1371/journal.pone.0015812

“Cause of death affects racial classification on death certificates”, by

Andrew Noymer, Andrew M. Penner, and Aliya Saperstein

**Detailed homicide and legal intervention ICD9 codes**

Homicide (ICD9 E960-E969) and legal intervention (E970-E978) are typically reported together in vital statistics, and given the relative dearth of legal interventions, are often referred to simply as homicides, a precedent that we have followed. Thus, any death that is classified as one of the following causes is considered to be a “homicide” in our analysis. We note also that we ran all models using the homicide codes only, without legal intervention, and the results were similar (not shown).

ICD9 codes for homicide and legal intervention:

(E960-E969) Homicide and injury purposely inflicted by other persons

E960 Fight, brawl, rape

E961 Assault by corrosive or caustic substance, except poisoning

E962 Assault by poisoning

E963 Assault by hanging and strangulation

E964 Assault by submersion [drowning]

E965 Assault by firearms and explosives

E966 Assault by cutting and piercing instrument

E967 Child battering and other maltreatment

E968 Assault by other and unspecified means

E969 Late effects of injury purposely inflicted by other person

(E970-E978) Legal intervention

E970 Injury due to legal intervention by firearms

E971 Injury due to legal intervention by explosives

E972 Injury due to legal intervention by gas

E973 Injury due to legal intervention by blunt object

E974 Injury due to legal intervention by cutting and piercing instruments

E975 Injury due to legal intervention by other specified means

E976 Injury due to legal intervention by unspecified means

E977 Late effects of injuries due to legal intervention

E978 Legal execution

**Next of kin classification in discordant cases**

This study examines patterns of racial classification discordance by comparing two different descriptions of the decedent: from a death certificate, and a proxy respondent survey. Our hypotheses focus on the racial distribution from official statistics that might result from racially stereotyped causes of death. We do not have specific predictions about the direction of corresponding next-of-kin classifications; however, some readers may be interested to know the overall distribution of such cases. Next of kin classifications vary widely when they do not agree with the death certificate classification. Table S1 reports the next of kin classification for all cases where next of kin and death certificate classifications are discordant.

**Models with sample restrictions**

In Table 1 of the main article, the sample sizes for Models 1–4 (estimating classification as American Indian when cause of death is cirrhosis) and Models 5–8 (estimating classification as Black when cause of death is homicide), are not consistent across each set of models. As described in the note to Table 1, this is a consequence of “perfect prediction” in the logistic regression model (e.g., there were no American Indians in certain occupational or income categories), which necessitates observations being dropped. We also estimated both sets of models using only the cases that are used in the final models for each cause of death (see Table S2). The results in Table S2 are the same as those in Table 1 in terms of statistical significance and substantive interpretation; thus, the results are not an artifact of changing sample size across the models.
